# Population-based screening for familial hypercholesterolaemia

**DOI:** 10.1515/medgen-2026-3033

**Published:** 2026-11-06

**Authors:** Christian Schlein, Sharlaine Y. L. Piel-Behrendt

**Affiliations:** University Medical Center Hamburg-Eppendorf Institute of Human Genetics Martinistr. 52 20246 Hamburg Germany; University Medical Center Hamburg-Eppendorf Institute of Human Genetics Hamburg-Eppendorf Germany

**Keywords:** population-based screening, familial hypercholesterolaemia, LDL cholesterol, genetic testing, early intervention

## Abstract

Familial hypercholesterolaemia (FH) is among the most common monogenic disorders, affecting at least 1 in 250 individuals. Lifelong elevation of LDL cholesterol from birth can cause accelerated atherosclerosis and premature cardiovascular disease. However, the vast majority of affected children (and adults) remain undiagnosed. Population-based childhood screening, combining LDL cholesterol measurement with genetic testing, enables early diagnosis and treatment. This review summarizes evidence on childhood FH screening, including disease burden, treatment efficacy, diagnostic performance, and economic considerations. Although no randomized trial has compared screened and unscreened populations, extensive indirect evidence supports the benefit of early detection and lipid-lowering therapy.

## 1 Introduction

Familial hypercholesterolaemia (FH) is among the most prevalent monogenic disorders worldwide. Heterozygous FH (HeFH) affects approximately 1 in 250 to 1 in 500 individuals [1], translating to an estimated 140,000 to 280,000 affected adults and 28,000 to 55,000 affected children and adolescents in Germany alone (calc. from [2]). The condition follows autosomal dominant inheritance and is caused predominantly by pathogenic variants in the LDL receptor gene (*LDLR*, 80–90 % of cases) with additional contributions from *APOB* (~ 5 %) and gain-of-function variants in *PCSK9* (1–2 %) [3]. Although variable expressivity is observed, individuals with FH usually exceed the recommended threshold for low-density lipoprotein cholesterol (LDL-C) of 116 mg/dL for healthy adults [4] already in early childhood, with LDL-C commonly above 190 mg/dL [5]. Of note, homozygous or compound-heterozygous pathogenic variants in FH-associated genes cause biallelic FH (global prevalence estimated 1:250,000 to 1:360,000) with LDL-C levels often above 400 mg/dL [6]; a comparable phenotype can also arise from digenic inheritance, most commonly as double heterozygosity in two FH-associated genes [7].

Lifelong exposure to elevated LDL-C drives accelerated atherogenesis from early childhood. Untreated men with FH carry a greater than 50 % risk of a fatal or non-fatal cardiovascular event by age 50, for untreated women, the risk exceeds 30 % by age 60 [8]. Autopsy and imaging studies have demonstrated that atherosclerotic lesions begin already in childhood, with their extent correlating with cholesterol levels [9, 10]. Carotid intima-media thickness is already measurably increased in children with FH before age 10 [11, 12]. Of note, a 20-year follow-up of the Dutch paediatric statin trial demonstrated that children with FH who commenced statin therapy in childhood had a progression of carotid intima-media thickness comparable to their unaffected siblings and cardiovascular event rates lower than their affected, later-treated parents [13], emphasizing the importance of early diagnosis and intervention.

Despite its high prevalence and the availability of effective treatment, FH remains vastly underdiagnosed. In most countries, fewer than 5 % of affected individuals are identified [14]. Germany lacks a systematic screening program, and the existing optional test regarding cholesterol levels at the German J1 adolescent health check at age 12 – 14 is utilized by only a minority of the population. Further, although the public insurance-paid health check from age 35 including LDL-C measurement can also identify individuals at risk, however, as LDL-C is already elevated from early childhood, affected individuals accumulate a high cumulative LDL-C burden over the first decades of life, driving premature atherosclerosis well before the age of first screening (Fig. 1a,b). Thus, the question of how to identify affected children before irreversible vascular damage occurs and reflects an important, yet unsolved question.

This work was designed as a narrative review with the aim to summarize the rationale, evidence, and challenges of population-based FH screening in childhood. The literature search, conducted between April 2026 and May 2026, was comprehensive, and updated in June 2026, ensuring the inclusion of the most up-to-date information during the revision. Literature research was conducted in PubMed, Scopus, and Web of Science using the terms “familial hypercholesterolaemia”, “FH”, “hyperlipidaemia” or “dyslipidaemia” and “screening”, “population-wide screening”, “population-based screening”, or “universal screening” and “childhood” or “children”. Articles published in 2020 – 2026 were prioritized to reflect the timeliness of data and clinical relevance of information.

**Figure:**
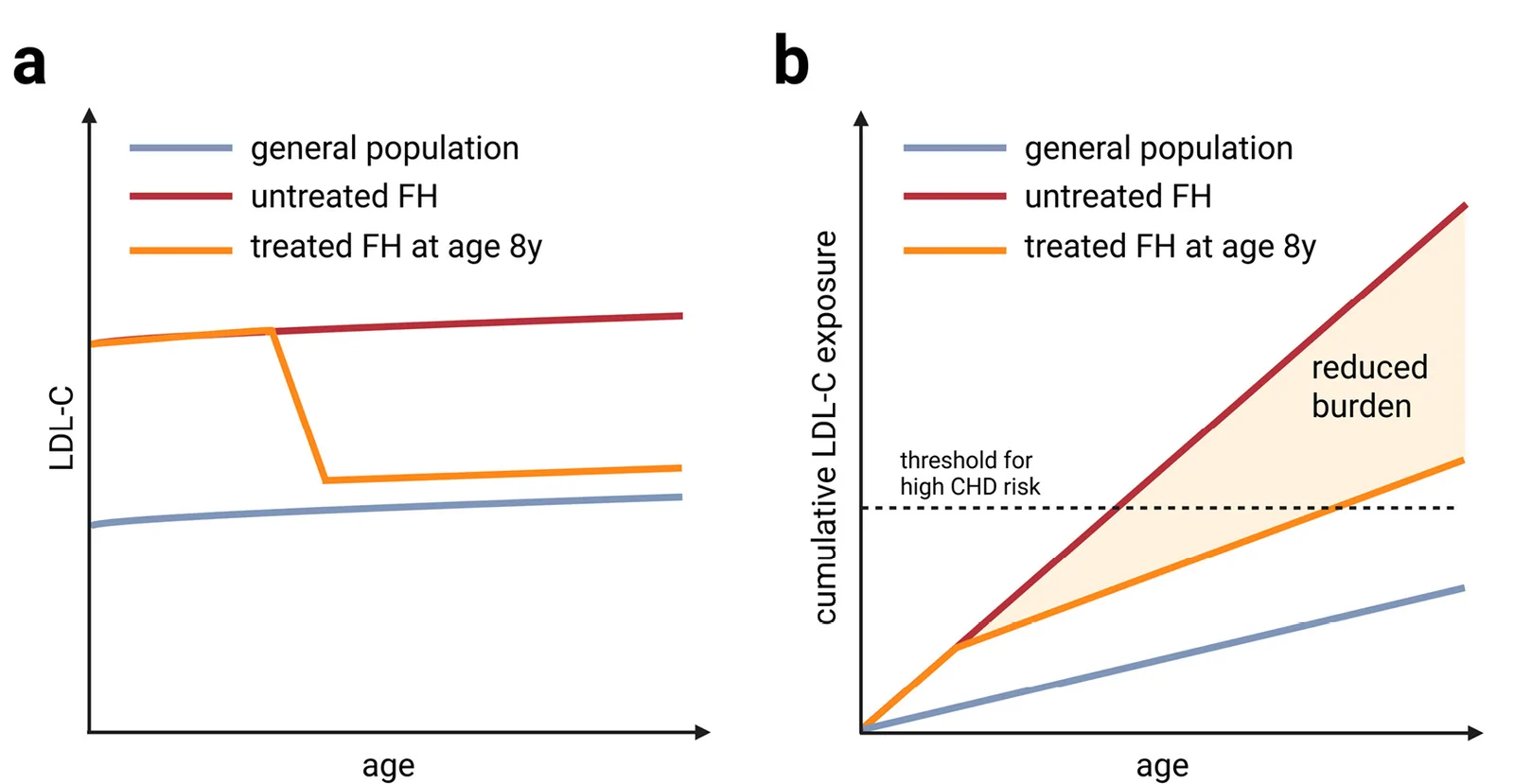
**Figure 1: Rationale for early identification and treatment of familial hypercholesterolaemia (FH).** **(a)** Schematic LDL-C levels over the life course. In the general population (blue), LDL-C rises gradually with age. In FH, LDL-C is markedly elevated from early childhood (red and orange). Without treatment, it remains high throughout life (red). Initiation of lipid-lowering therapy in childhood (orange; example shown for treatment started at 8 years of age) lowers LDL-C substantially. **(b)** Corresponding cumulative LDL-C exposure (“cholesterol-years”). Because untreated FH (red) combines high LDL-C with lifelong duration, cumulative exposure accrues rapidly and far exceeds that of the general population (blue). Early treatment (orange) markedly attenuates this. The shaded area (“reduced burden”) indicates the cumulative LDL-C exposure avoided by initiating therapy in childhood rather than leaving FH untreated. The dashed line indicates a conceptual threshold of cumulative LDL-C exposure above which coronary heart disease (CHD) risk increases substantially (per illustration, excluding absolute values). Created in BioRender. Schlein, C. (2027), https://BioRender.com/k3lfm1c.

## 2 Screening strategies for FH: an overview

Three principal strategies for the early identification of FH in children have been proposed and evaluated in clinical practice and guidelines: Cascade screening, selective screening, and population-based screening. All strategies include lipid measurements followed by second-step genetic testing. Of note, a population-based approach integrating FH screening into newborn screening, analogous to the genetic testing for the most common pathogenic *CFTR* variants in cystic fibrosis within the German extended newborn-screening programme [15], is currently not broadly discussed. LDL-C levels in newborns discriminate poorly between affected and unaffected individuals and only reliably identify FH from approximately one year of age onwards [16].

### 2.1 Cascade screening

The principle of cascade screening is that, once an index patient has been diagnosed with FH, their close biological relatives are systematically offered lipid measurement and where possible, targeted genetic analysis for the familial variant. Because FH follows autosomal dominant inheritance, each first-degree relative has a 50 % probability of carrying the same pathogenic variant. By extending the search to second- and third-degree relatives, a single confirmed diagnosis can uncover multiple affected family members who would otherwise remain undetected. The NICE guideline on identification and management of FH systematically evaluated cascade screening and recommended lipid measurement combined with DNA testing, and a subsequent UK Health Technology Assessment concluded that cascade testing from genetically confirmed index cases represents a cost-effective strategy for identifying individuals with FH [17]. Cascade screening identifies FH in 30 to 60 % of tested relatives, with a number needed to test of only two to three, meaning that for every two to three relatives tested, one new FH case is found. Detection rates are highest when healthcare professionals contact relatives directly rather than relying on the index patient to inform the family, and when genetic testing is used for confirmation after increased LDL-C was measured [18].

Evidence for cascade screening is provided by the Netherlands, where FH identification was pioneered through a comprehensive cascade-screening program lead by a FH cascade screening foundation, that achieved a 71 % detection rate [14]. However, cascade screening has a fundamental structural limitation: it can only be initiated from a known index case. This creates a circular problem that index cases have to be identified. In Germany, efforts have been made to establish a systematic cascade-screening infrastructure but still, the vast majority of FH cases remain undiagnosed [19]. In the absence of a sufficient discovery rate of index cases, cascade screening alone cannot close the diagnostic gap. It reflects an essential complement to, but not a first line strategy for identifying new cases independently of family history.

### 2.2 Selective screening

Selective screening restricts lipid testing to children considered at higher risk based on their family history, usually a parent or grandparent with known hypercholesterolaemia or premature coronary heart disease. The rationale is that FH clusters in families and thus, a positive family history should enrich the screened population for affected children and improve efficiency.

In practice, however, this approach performs poorly. Many families are unaware of their blood lipid levels or the cardiovascular history of their family, and premature cardiovascular events of relatives may not yet have occurred at the time of the screening visit. The US CARDIAC study illustrates this limitation: Among ~20,000 schoolchildren, the prevalence of hypercholesterolaemia was nearly identical in children with a positive family history (8.3 % at an LDL-C threshold of 130 mg/dL) and those without (9.5 %), demonstrating that family history failed to distinguish high-risk from low-risk children [20]. Selective screening based on family history alone would therefore miss a substantial number of affected children.

### 2.3 Population-based screening

The limitations of both cascade and selective screening point toward a third strategy: offering lipid measurement to all children within a defined age range, regardless of family history or prior diagnoses in the family. In population-based screening models, an LDL-C measurement is performed during a routine paediatric visit. Children whose LDL-C exceeds a predefined threshold are then referred for molecular genetic testing to distinguish monogenic FH from other causes of elevated cholesterol.

Slovenia became the first country to implement population-based, opt-out cholesterol screening for FH in children, which was integrated into the pre-school health check at age 5 [21, 22] (see Table 1). Approximately 20,000 children are screened annually within a structured system connecting specialized lipidology centres, genetic counselling, and a digital data registry. Feasibility and population acceptance have been reported [23]. In Germany, the existing U-Vorsorge system of scheduled preventive health checks for children provides a natural infrastructure for integration into clinical practice. Visits such as the U9 (around age 5) already include blood-sampling capability and reach over 95 % of the targeted population without additional clinic visit [24]. The genetic confirmation step applies only to the small minority of children with elevated LDL-C results, typically around 5 % or less of those screened, depending on the chosen threshold. For these children, targeted analysis of the FH-associated core genes can provide a molecular genetic diagnosis. This two-step design of using a broad biochemical pre-selection test followed by targeted genetic confirmation combines the population-based range needed to identify previously unknown cases with the diagnostic precision of molecular genetics.

## 3 The evidence base for population-based FH screening

The decision to introduce a population-based screening program must rest on a coherent chain of evidence: 1) The condition must be common and the results need a consequence to justify systematic detection. 2) A reliable test must exist, 3) early identification must translate into better outcomes than later or opportunistic diagnosis and 4) the potential harms must not outweigh the benefits. For FH, each link in this chain is supported by evidence, though not all links are equally strong, and important uncertainties remain.

### 3.1 Disease burden and natural history

As outlined, FH is among the most common monogenic disorders and untreated, FH causes premature atherosclerotic cardiovascular disease [5, 25] associated to potentially fatal outcomes. Importantly, the atherosclerotic process starts before adulthood [9–12]. The damage accumulates continuously from birth and by the time symptoms appear, it is often advanced and partially irreversible. At the same time, there is clear evidence that this disease progress can be altered. Population-level registry data from Denmark show that the life expectancy gap between individuals with FH and the general population has narrowed from 22 years in 1978 to a statistically non-significant (p=0.16) one year in 2021, most likely as a consequence of improved diagnosis rates, wider statin use and better emergency therapy of cardiovascular events [26]. However, corresponding coronary heart disease (CHD) still occurred 7 years earlier (from 20 years in 1978) in individuals with FH, underlining that the individuals likely still experience more CHD-associated events. This emphasizes that patients, once identified, benefit substantially from therapy but earlier diagnose still may lead to further reductions in CHD-related events and outcome and thus, diagnosing FH is of high importance for the individual health and health system.

### 3.2 Effectiveness of early treatment

The central question for any screening program is whether earlier detection leads to better outcomes than clinical diagnosis at a later stage. For FH, this question cannot be answered by a randomized trial of screening versus no screening as the decades of latency between childhood diagnosis and cardiovascular events make such a study impractical, and arguably unethical given what is already known.

The available evidence is therefore indirect but substantive. Statins reliably reduce LDL-C by approximately 30 to 40 % in children with FH, with an acceptable short-term safety profile established in randomized controlled trials [27]. Long-term tolerability and adherence data from specialized centres are encouraging [28]. Additional lipid-lowering agents, such as ezetimibe and PCSK9 inhibitors, are available for non-adult patients who do not reach therapeutic targets with statins alone [29].

Importantly, simply treating any elevated LDL-C would be a possibility, however, FH confers cardiovascular risk beyond what the LDL-C level alone would predict [30, 31] and given the fact that carriers of pathogenic variants causing FH can also have periods of LDL-C within the range, FH may display reduced penetrance and variable expression. However, at any given LDL-C concentration, individuals with monogenic FH have a significantly higher risk of coronary artery disease compared to individuals with the same LDL-C from non-monogenic causes [30, 31]. This excess risk may be attributed to the cumulative lifetime burden of elevated LDL-C beginning at birth (Fig. 1a,b), rather than a gradual rise in midlife. It also can be seen as an indicator that FH patients require more aggressive lipid-lowering targets and earlier treatment initiation than would be necessary by a single cross-sectional cholesterol measurement. However, more studies investigating the optimal treatment targets in FH in the different stages of life are needed.

A study from the Netherlands conducted a 20-year follow-up study of statins in children with FH [13]. In the treated cohort, only one single cardiovascular event had occurred by age 32, compared to 41 events (26 %) among their affected parents before age 40. Additionally, the progression of carotid intima-media thickness in the treated children (now: adults) was indistinguishable from that of their unaffected siblings. Of note, the study was retrospective, the parent comparison group was not simultaneously randomized, and selection bias toward more severely affected families is likely. However, it shows that associations between early treatment and cardiovascular outcome can be detected and these data, despite their observational limitations, represent the strongest available evidence that early identification and treatment fundamentally alter the progress of FH.

Mendelian randomization studies provide further complementary support. Genetic variants associated with lifelong lower LDL-C show a roughly threefold greater reduction in coronary heart disease risk per mmol/L LDL-C lowering than statin therapy initiated in middle age [32]. In sum, this emphasizes that in FH, both the cholesterol concentration and the time duration of exposure drive risk and that intervening early in life reflects a benefit window that cannot be recovered later, underlining the importance for early FH detection (Fig. 1a,b).

### 3.3 Performance of the screening test

A screening program needs an ability to distinguish affected from unaffected individuals at acceptable rates of error. For FH screening, LDL-C measurement serves as the initial filter, and genetic testing can provide confirmation.

Wald et al. [33] screened 10,095 children aged 1 to 2 years in UK general practices using total cholesterol (see Table 1), followed by genetic testing from the same sample (analysis of hot-spot mutations in *LDLR* (46), *APOB* (1), and *PCSK9* (1); if the beforementioned were uninformative, additional *LDLR*, *APOB* and *PCSK9* sanger sequencing plus *LDLR* MLPA). An analysis by IQWiG [34] reported a sensitivity of 54.1 % and specificity of 99.3 % for detecting genetically confirmed FH at a threshold of 5.95 mmol/L (230 mg/dL) total cholesterol. This means that roughly half of affected children were missed, but very few unaffected children were falsely flagged. Futema et al. [35] measured LDL-C directly in 5,083 ten-year-old children (see Table 1), of whom a random group of 30 % received genetic testing (whole-genome or gene panel analysis). The analysis by IQWiG [34] reported higher sensitivity (66.7 %) and perfect specificity (100 %) at a threshold of 4.25 mmol/L (164 mg/dL) LDL-C, suggesting that LDL-C is a more suitable screening parameter than total cholesterol.

**Table 1:** **Selected FH screening studies in Europe.** Sens. = sensitivity, Spec. = specificity.

**Screening study**	**Location**	**Design**	**Screening population**	**Age at screening**	**Cholesterol threshold**	**Test performance**
Child–Parent Familial Hypercholesterolaemia Screening in Primary Care [33]	United Kingdom	Opt-in, all received genetic testing	10,095 children	1 – 2 years	Total cholesterol: 5.95 mmol/L (230 mg/dL)	Sens.: 54.1 % Spec.: 99.3 % [34]
Screening for familial hypercholesterolaemia in childhood: Avon Longitudinal Study of Parents and Children (ALSPAC) [35]	England	Opt-in, 30 % (random group) received genetic testing	5,083 children total, 1,497 children completed genetic testing	10 years	LDL-C: 4.25 mmol/L (164 mg/dL)	Sens.: 66.7 % Spec.: 100 % [34]
Universal screening for familial hypercholesterolaemia in children: the Slovenian model [21, 22]	Slovenia	Opt-out, genetic testing only above LDL-C threshold	154,658 children (born 2006-2013; 15,109 opt-outs excluded)	5 years, at preschool check	LDL-C: 3.5 mmol/L (135 mg/dL)	Combined data of Slovenia and Fr1dolin-trial of 945 children with completed genetic analysis: Sens.: 90.5 % Spec.: 55.3 % [42]
The Fr1dolin-trial [36, 37]	Germany, Lower Saxony and Hamburg	Opt-in, genetic testing only above LDL-C threshold	15,009 children	2 – 6 years	LDL-C: 3.5 mmol/L (135 mg/dL)	
The VRONI study [38–41]	Germany, Bavaria, Lower Saxony and Hamburg		Over 40,000 children	5 – 14 years	3.34 mmol/L (130 mg/dL)	PPV: 15 – 16 % [34]; 17 % [41]

Two major pilot studies are generating Germany-specific data: The Fr1dolin study in Lower Saxony and Hamburg combined FH and type 1 diabetes screening in 15,009 children aged 2 to 6 years, offering genetic testing for FH at an LDL-C threshold of 3.5 mmol/L (135 mg/dL) [36, 37], and the ongoing VRONI study in Bavaria, Lower Saxony and Hamburg has screened over 40,000 children aged 5 to 14 years through paediatricians during routine U9 to J1 health checks, using a lower LDL-C threshold of 3.34 mmol/L (130 mg/dL – or 93^rd^ percentile) to trigger genetic testing [38–41] (see Table 1). Genetic testing was conducted using a next-generation-sequencing FH panel in both Fr1dolin and VRONI. Both studies share the limitation that children below the screening threshold were not genetically tested, excluding them for a reliable determination of sensitivity and specificity for the whole screening population [34]. On the other hand, children with LDL-C levels below 130 mg/dl are currently not considered for medical treatment. Apart from that, a combined study with data from the Fr1dolin study and data from the Slovenian program (see: 2.3 population-based screening) calculated the test performance within the group of children that completed genetic testing (total: 945 children). Sensitivity of 90.5 % and specificity of 55.3 % at an LDL-C threshold of 3.5 mmol/L (135 mg/dL) for confirming FH were reported [42] (see Table 1), but only apply to the cohort that received genetic testing therefore cannot be used to evaluate the true performance of the whole two-step screening process [34]. Due to the same methodological limitation, only the positive predictive value (PPV) can be calculated for the VRONI study. Within the first 19,000 screened participants, the PPV was 15 to 16 % [34] for those children who received genetic testing, and more recently published data including 25,431 screened participants reports 17 % [41], meaning that approximately 1 in 6 children with an elevated LDL-C had genetically confirmed FH at the lower threshold of 3.34 mmol/L (130 mg/dL). Of note, the fraction of individuals with FH-causing variants increased gradually across the spectrum of LDL-C serum concentrations, reaching 78.6 % above 5.17 mmol/L (200 mg/dL). The comparison of the test performances at the reported LDL-C thresholds, that were differentially defined, and the recently published positive correlation of LDL-C serum concentrations and PPV [41], illustrate the fundamental trade-off in threshold selection: A lower cut-off captures more true cases but flags more children for unnecessary follow-up, a higher cut-off reduces false positives but misses more affected individuals. Because children below the threshold were not genetically tested in either German pilot study (in both VRONI and Fr1dolin), true sensitivity and specificity remain unknown in the German setting. Nevertheless, both studies provide essential data on feasibility, positive predictive value, logistical requirements for a potential national program and were excellently implemented in daily routines of caretakers showing a possibility of the implementation in Germany is given.

### 3.4 Acceptability, harms, and open questions

Qualitative studies from multiple countries consistently report high interest of the parents to participate in FH screening [43–45]. Parents value the possibility of early detection and prevention, and practical experience from the VRONI study confirms that German paediatricians find the process feasible within routine practice [38, 39], and that the combined biochemical and genetic screening for FH in children is overall feasible and efficient [41].

The most frequently raised concerns include psychological distress from a cholesterol-related diagnosis in childhood, the risk of “labelling” children as chronically ill, potential implications for insurance or career and family conflict arising from the identification of an inherited condition. It should be noted, however, that systematic data on psychosocial outcomes in the specific context of childhood FH screening are scarce, and the concerns at this point remain largely theoretical.

An unresolved issue is the path in the health system for children with elevated LDL-C who do not carry a monogenic FH variant. At an LDL-C threshold of 3.34 mmol/L (130 mg/dL), approximately 1 in 14 or 15 of screened children will have an elevated result, but only about 1 in 6 of these will have genetically confirmed FH [34, 41]. For the majority, who have multifactorial or polygenic hypercholesterolaemia [31], no data exist on potential benefits of pharmacological treatment in childhood. How these families should be counselled and followed remains an unresolved question.

Health-economic evidence is similarly inconclusive. A systematic review of 14 modelling studies found no consistent evidence on cost-effectiveness. The results were highly sensitive to assumptions about test performance, treatment adherence, and healthcare system costs [46]. A recent US model concluded that population-based screening at age 10 was not cost-effective by American thresholds [47], but US cost structures are not transferable to the German health insurance system. Importantly, the largest cost in this model was due to a dedicated patient visit for the initial LDL-C measurement. A UK model found favourable cost-effectiveness when childhood screening was combined with reverse cascade testing of parents [48]. No robust German-specific analysis exists to date.

## 4 The human genetics perspective

In the proposed two-step screening algorithm, genetic testing serves as the confirmatory step after biochemical pre-selection, transforming a population-level lipid measurement into a precise molecular diagnosis.

### 4.1 Genetic testing in the screening cascade

Panel-based sequencing of *LDLR*, *APOB*, *PCSK9*, and for suspected autosomal recessive cases *LDLRAP1*, is well established in diagnostic genetics laboratories [49]. Also, experience from the VRONI study demonstrated that panel-based genetic testing is superior to hotspot variant screening and feasible in population-based FH screening [41]. High-throughput analysis, as already implemented for newborn screening programs, could be adapted for FH screening.

### 4.2 Variant interpretation

A substantial challenge lies in variant classification. Over 2,000 variants have been reported in *LDLR* alone, many classified as variants of uncertain significance (VUS). In a screening context, VUS results can create uncertainty for families and clinicians as they neither confirm nor exclude FH and may trigger unnecessary anxiety or, conversely, false reassurance. Systematic application of ACMG classification criteria and analysis of publicly available variant data have highlighted significant discrepancies between database annotations and evidence-based classifications [50]. Robust variant databases, functional assays, and standardized classification criteria are essential prerequisites for reliable screening. This is a major challenge, which needs structural efforts for supporting systematic scientific functional analyses in centralized and standardized environments.

### 4.3 Genotype-phenotype discordance

The key reason why identifying FH via molecular genetic testing rather than simply treating any elevated LDL-C matters, is the already mentioned (see 3.2) extensive height of the FH-associated cardiovascular risk [30, 31]. Although a substantial amount of FH carriers displays only moderately increased LDL-C serum concentrations, cumulative lifetime burden of elevated LDL-C beginning at birth causes a lifelong increasing cardiovascular risk that demands earliest treatment.

In detail, a recent German cross-sectional study found that only 48 % of genetically confirmed FH carriers had a severe form of hypercholesterolaemia defined by LDL-C ≥ 190 mg/dL [51]. Consequently, 52 % of genetically confirmed FH carriers would be missed by a purely biochemical risk assessment with a threshold of LDL-C ≥ 190 mg/dL, yet they carry the same lifelong genetic predisposition and a substantial cumulative vascular burden. Of note, the VRONI study recently published a prevalence of FH-causing variants in German children of 1 in 90, with a bias-adjusted predicted prevalence of 1 in 163 [41], highlighting the gap across the prevalence of clinically overt hypercholesterolemia of 1 in 250 to 1 in 500 individuals [1]. Therefore, genetic testing is therapeutically relevant.

### 4.4 Legal and ethical considerations

The German Genetic Diagnostics Act (Gendiagnostikgesetz, GenDG) distinguishes between diagnostic genetic testing in symptomatic individuals and predictive testing in asymptomatic persons. In the screening context, a child with elevated LDL-C undergoes diagnostic testing to clarify the cause of an observed abnormality. However, cascade testing of asymptomatic siblings based on a proband’s result constitutes predictive testing, requiring genetic counselling by a qualified physician under GenDG. These regulatory requirements must be considered when designing screening infrastructure.

### 4.5 Reverse cascade screening

An often-underappreciated benefit of paediatric FH screening is its potential for reverse cascade testing. A study from the United States found that each child identified with FH has, on average, one affected parent and 3.55 ± 1.87 additional affected family members who can be identified through systematic follow-up [52]. Although these numbers cannot be directly transferred to the German population, paediatric screening may serve as a gateway to identifying undiagnosed adults at immediate cardiovascular risk and thus, would serve greater public health.

## 5 Unresolved questions

For childhood FH screening, several important questions remain open, and not all expert bodies agree that the available evidence justifies implementation. The main contra argument is that there is no direct evidence that screening children for FH improves cardiovascular outcomes. No trial has ever compared screened with unscreened populations and measured heart attacks or deaths decades later. Both the IQWiG rapid report [34] and the US Preventive Services Task Force [53] reached the same conclusion that the direct evidence would be insufficient, because no study compared screened vs unscreened populations for cardiovascular complications. However, a randomized trial with cardiovascular endpoints would require decades of follow-up, and withholding a potentially beneficial intervention from the control group raises its own ethical problems.

Beyond the evidence question, it was argued that Germany should prioritize establishing effective cascade screening before adding a population-based program. This position carries weight given that the Netherlands achieved high detection rates through cascade screening alone, without population-based childhood testing. The Dutch indeed are encouraging, but generating sufficient amount of index cases for implementing a broad cascade screening has failed over the last decades in Germany, given differences in the health care system. Thus, it is likely that modifying this branch will not result in the requested rates of diagnosis alone.

Further, false-positive biochemical results were discussed: At an LDL-C threshold of 3.34 mmol/L (130 mg/dL), roughly 1 in 14 or 15 children will be flagged, but only about 1 in 6 of these will actually have monogenic FH [34, 41]. For children with multifactorial hypercholesterolaemia, there is currently no evidence-based pharmacological treatment in childhood, yet their families will have been informed of an abnormal cholesterol result with not yet certain clinical significance. Lifestyle counselling is a reasonable possibility but a clear follow-up pathway is needed. Further, also systematic data on psychosocial effects how a childhood FH diagnosis or an inconclusive cholesterol result affects children and their families should be initiated to avoid anecdotic evidence about generating anxiety by screening.

The optimal LDL-C cut-off for the initial biochemical screen has not yet been established. Lower thresholds detect more FH cases but generate more follow-up visits and genetic tests for children who ultimately do not have monogenic disease. Neither the VRONI nor the Fr1dolin study tested children genetically below their respective cut-offs, and thus, the underlying sensitivity and specificity in the German population remain unknown. Further, no health-economic analysis specific to the German statutory health insurance system exists and models from other countries have produced contradictory results that cannot simply be transposed.

The core argument of pro-screening colleagues counter that waiting for perfect evidence dooms another generation of children with FH to delayed diagnosis and preventable cardiovascular disease. They note that the evidence gap is structural: In their view, the strength of the indirect evidence chain, combined with the feasibility data from pilot programs, justifies a carefully monitored implementation rather than indefinite rescheduling.

Both positions share one essential recognition that any screening program must be accompanied by structured evaluation. Whether this takes the form of a cluster-randomized trial, or a register-based implementation study with predefined quality indicators, the principle is the same – screening should be rolled out as a program that generates the evidence needed to confirm or correct its own assumptions [54].

## 6 Conclusion and outlook

FH is common, consequential, treatable, and detectable in childhood. Although randomized screening trials are lacking, broad indirect evidence demands early action. Germany is well-positioned to take this path within the U-Vorsorgesystem, with a feasible two-step screening model of LDL-C measurement followed by genetic confirmation.

What is needed now is a structured path toward implementation that addresses the legitimate concerns raised within the discussion of professionals. This requires a German-specific health-economic evaluation, a standardized screening protocol, and a management pathway for children with elevated LDL-C but no monogenic diagnosis. Further, continuous evaluation is needed within a national FH registry. Lastly, as each diagnosed child enables identification of affected relatives at immediate cardiovascular risk, reverse cascade screening must not be forgotten.

The evidence for FH screening will never be complete before a decision is made – but the sum of epidemiological data, treatment evidence and pilot program experience, together with declining genetic testing costs, creates a unique window of opportunity. 
